# Interrelationship between dental clinicians and laboratory technicians: a qualitative study

**DOI:** 10.1186/s12903-023-03395-z

**Published:** 2023-09-20

**Authors:** Eman H. Ismail, Dalya Al-Moghrabi

**Affiliations:** 1 Department of Clinical Dental Sciences, College of Dentistry, Princess Nourah bint Abdulrahman University, Riyadh, Saudi Arabia; 2 Department of Preventive Dental Sciences, College of Dentistry, Princess Nourah bint Abdulrahman University, Riyadh, Kingdom of Saudi Arabia

**Keywords:** Dental laboratories, Allied health personnel, Patient-centered care, Technology, Communication, Leadership

## Abstract

**Background:**

Effective communication among members of the dental team is essential for the delivery of high-quality dental care. However, an in-depth understanding of issues concerning the interrelationship between dental clinicians and laboratory technicians has not been previously undertaken. Therefore, the aim of the study was to explore factors influencing the interrelationship between dental clinicians and laboratory technicians.

**Methods:**

Semi-structured interviews were conducted with dental clinicians and laboratory technicians using purposeful snowball sampling. Two trained researchers conducted the interviews based on a pre-piloted topic guide. The interviews were conducted via video conferencing platform, audio-recorded, and transcribed verbatim. Field notes were taken during the interviews. Framework Methodology was used to analyze the data.

**Results:**

A total of 20 dental clinicians and laboratory technicians were interviewed. The average interview duration was 37 min. Participants mainly reported negative encounters and highlighted the importance of training and exposure, collaborative learning, and alignment of expectations of both parties in terms of cost of laboratory work, turnaround time, and patient preferences. The relationship between dental clinicians and laboratory technicians depends largely on effective teamwork dynamics and open communication channels. Increased workload, workforce shortage, availability of digital systems, management policies, and financial challenges were emphasized as organizational factors affecting the interrelationship between both groups. Participants highlighted the importance of shadowing, mentorship, education courses, joint discussions, patient-technician rapport, and adoption of digital technology for fostering collaborative practices between the professions.

**Conclusions:**

A multitude of factors influencing the dental clinician-laboratory interrelationship at individual, interpersonal and organizational levels were identified. This study highlights the need to build a transformative relationship underpinned by mutual trust and respect. Such a collaborative relationship will facilitate optimal patient care and successful treatment outcomes. The outcome of this study can help stakeholders identify solutions for enhancing the interrelationship among the dental team, to ultimately improve patient care and efficiency of dental services.

## Background

Interrelationship among the dental team is vital for the efficient delivery of dental services, and any disruption is likely to be consequential. Notwithstanding this, the issue of miscommunication between dental clinicians and laboratory technicians is a globally recognized issue [1–8]. It has been perpetually documented in publications for almost 5 decades [1–9], despite the advent of new technologies and evolving communication methods. Furthermore, descriptions such as “love-hate relationship” and “friends or enemies?” have been used to describe the nature of this complex relationship [10, 11]. Some studies have reported that more than one-quarter of dental laboratory technicians experienced a lack of involvement in the dental team [12], and less than half of them felt valued as members of the team [13]. For example, in a recent survey, only 31% of technicians felt they played an integral role in prosthesis fabrication [7]. In another survey, one-third of dental technicians working in commercial laboratories felt that their communication with dentists was neither welcomed nor encouraged [6]. Therefore, there is a critical need for a detailed understanding of the issues undermining this unique interprofessional relationship.

Intuitively, clinician and laboratory technician encounters are heavily reliant on effective and purposeful teamwork. Notwithstanding this, the social aspects of interpersonal communication between the two groups were overlooked in previous studies, with emphasis given to technical aspects [5, 8, 14]. For example, receiving inadequate prescriptions, substandard impressions, and missing dental records [5, 8, 14]. Moreover, some reports suggest that clinicians rely primarily on technicians to make decisions regarding appropriate material selection for fixed partial dentures [5]. Contrary views concerning the perceived leadership role have been documented; 61% of prosthodontic specialists in one study felt that clinicians had the lead role in treating patients with fixed-implant prostheses [15]. However, only 15% of dental laboratory technicians felt this was a clinician’s role [15]. Notably, most of the studies were questionnaire-based, which may have been biased by the researchers' preconceptions rather than being a true reflection of the participants’ voices. Therefore, detailed understanding of the problem from the participants’ perspectives remains unclear.

The documented consequences of miscommunications between dental clinicians and laboratory technicians include remakes and the consequent associated revenue loss, delays, and dissatisfaction of the dental team and patients [16, 17]. Indeed, a recent qualitative study highlighted the importance of teamwork and feelings of involvement as determinants of job satisfaction for clinicians and dental laboratory technicians [18]. However, there is a paucity of research evaluating the barriers to effective communication among members of the dental team. Therefore, the aim of this study was to explore factors influencing the interrelationship between dental clinicians and laboratory technicians.

## Methods

Ethical approval was obtained from Princess Nourah bint Abdulrahman University (IRB log number: 21–0247). Purposeful snowball sampling was employed, in which participants nominated other participants from different regions and sectors in their professional group [19]. This study included dental clinicians from different specialties (restorative dentistry, orthodontics, prosthodontics, and pediatric dentistry) and laboratory technicians working in different sectors. The recruitment of participants from both groups continued until data saturation was achieved.

A participant information sheet was provided, and oral and written consent was obtained from all participants. Before participant recruitment, a topic guide was developed and piloted by two authors (EI and DA). Data obtained from the pilot interviews were not included in the analysis. The interviews covered aspects pertaining to previous experiences, causes of positive or negative encounters, communication dynamics, consequences, and potential solutions. Non-leading probing questions were used to capture detailed information. Demographic information, educational level, and work experience were recorded.

Both authors conducted the interviews via video calls using Zoom (San Jose, CA, USA). The interviews were audio-recorded and transcribed verbatim. The transcriptions were reviewed by one interviewer (EI) to check accuracy. Field notes were taken during the interviews, and qualitative data analysis was performed using Framework Methodology [20]. Additionally, coding was undertaken by two authors (EI and DA) using NVivo qualitative data analysis software (Release 1.7.1; QSR International Pty Ltd.).

Both interviewers (EI and DA) undertook training in qualitative research. Trustworthiness and rigor in the qualitative research were established through debriefing via joint discussions (EI and DA) to verify the interpretations of the participants’ voices. Additionally, prolonged engagement was undertaken with the participants to help them thoroughly describe their experiences. A member check was conducted following the data analysis to assess the accuracy of the findings.

## Results

Twenty participants were interviewed, including dental clinicians (*n* = 10) and laboratory technicians (*n* = 10) from different regions and work sectors in Saudi Arabia (Table 1). Of these, five participants owned dental clinics and/or laboratories. The years of experience were between 1–20 and 7–31 years for dental clinicians and laboratory technicians, respectively. Each interview lasted between 29 and 90 min (average: 37 min).

**Table 1:** General information of participants including demographics and clinical experience (*n* = 20)

Characteristics	Dental clinicians (*n *= 10)	Dental laboratory technicians (*n *= 10)	Overall sample (*n *= 20)
Gender			
Male	*n* = 5	*n* = 7	*n* = 12 (60%)
Female	*n* = 5	*n* = 3	*n* = 8 (40%)
Age			
20–30 y	*n* = 2	*n* = 2	*n* = 4 (20%)
31–40 y	*n* = 6	*n* = 3	*n* = 9 (45%)
41–50 y	*n* = 2	*n* = 3	*n* = 5 (25%)
51–60 y	*n* = 0	*n* = 2	*n* = 2 (10%)
Qualifications			
Diploma	*n* = 0	*n* = 3	*n* = 3 (15%)
Graduate	*n* = 2	*n* = 3	*n* = 5 (25%)
Postgraduate	*n* = 8	*n* = 4	*n* = 12 (60%)
Type of postgraduate program			
Local programs	*n* = 5	*n* = 4	*n* = 9 (45%)
Abroad	*n* = 5	*n* = 6	*n* = 11 (55%)
Work experience			
1–5 y	*n* = 2	*n* = 0	*n* = 2 (10%)
6–10 y	*n* = 2	*n* = 2	*n* = 4 (20%)
11–15 y	*n* = 4	*n* = 4	*n* = 8 (40%)
16–20 y	*n* = 2	*n* = 1	*n* = 3 (15%)
> 21y	*n* = 0	*n* = 3	*n* = 3 (15%)
Type of practice			
Hospital job	*n* = 6	*n* = 5	*n* = 11 (55%)
Private clinic	*n* = 2	*n* = 3	*n* = 5 (25%)
Academics	*n* = 2	*n* = 2	*n* = 4 (20%)
Region of workplace			
Central	*n* = 5	*n* = 4	*n* = 9 (45%)
Northern	*n* = 1	*n* = 0	*n* = 1 (5%)
Southern	*n* = 1	*n* = 1	*n* = 2 (10%)
Eastern	*n* = 1	*n* = 3	*n* = 4 (20%)
Western	*n* = 2	*n* = 2	*n* = 4 (20%)

*y* years

**Fig. 1:**
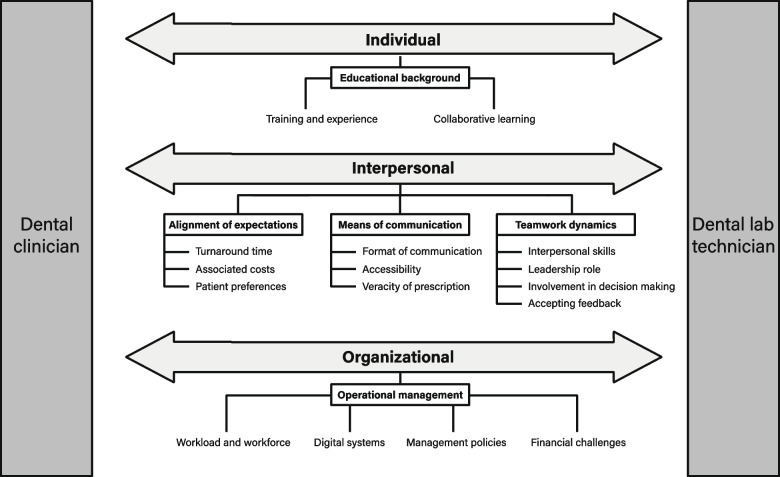
Factors influencing the interrelationship between dental clinicians and dental laboratory technicians

Several individual, interpersonal, and organizational factors influenced the interrelationship between dental clinicians and laboratory technicians (Fig. 1). Five themes emerged from the data: quality of education and acquired experience, alignment of expectations, teamwork dynamics, means of communication, and operational management (Fig. 1).

### Theme 1. Educational background

Differences in the types of techniques and materials used were perceived as a challenge that prevents adequate collaboration between dental clinicians and laboratory technicians. This was mainly attributed to disparity in the educational and training backgrounds of both groups. Furthermore, limited English language proficiency among some laboratory technicians was reported as a barrier to accessing the latest dental literature, resulting in dissimilar exposure to the latest research findings and up-to-date approaches between the groups.

Dental laboratory technicians reported that most of their undergraduate teaching was delivered by dental clinicians, which undermined their understanding of their role and technical skill requirements as dental technicians. Thus, most laboratory technicians lacked role models and mentors from within the profession, and some attributed this to the limited number of technicians in academia. They further explained that laboratory technology programs are usually siloed at different colleges, with no interaction among their counterparts in other dental programs. Hence, the educational needs and outcomes of the two programs were not considered to be aligned.

Dental laboratory technicians reported a scarcity of postgraduate programs and specialized courses, with the main source of learning occurring in workshops offered by materials companies. In orthodontics, clinicians reported many instances in which technicians were unaware of the different types of appliances and their components. Furthermore, the suboptimal hands-on skills of dental laboratory technicians were among the factors that restricted clinicians’ choice of dental laboratory technicians. Clinicians in this study were more comfortable with experienced and competent technicians. The important indicators of technician competency expressed by dental clinicians were the length of their careers and the monthly number of completed units or cases.

Both dental clinicians and laboratory technicians emphasized the necessity for robust collaborative education and knowledge sharing. The participants highlighted the importance of dental laboratory technician students shadowing clinicians in clinical settings to better understand some of the procedural steps and to develop empathy towards patients. Similarly, the participants agreed that exposing dental students to laboratory settings during their studies would enhance their appreciation of the diversity of available dental materials, time required to complete cases, and identification of errors in impressions. These interactions were believed to foster empathy between the two professions.

LT6: “Some [dental] students ask for their case to be ready tomorrow; they do not understand the lengthy steps that need to be followed.”

Several issues related to clinician knowledge were raised by technicians, including reliance on the technician to make material selection decisions due to clinicians being unfamiliar with details about certain types of materials.

LT4: “Clinicians ask us [technicians], ‘What do you recommend? Whatever material you recommend, go for it.’ Sometimes clinicians just say ‘E.max or VITA’ … VITA has 300 different types of materials.”

Dental laboratory technicians reported the need for guidance from dental clinicians and viewed them as a source of learning, especially early on in their careers. Reflecting on their work experiences, dental laboratory technicians appreciated joint discussions with treating clinicians and perceived them as having provided learning opportunities that helped them improve the quality of their work. Dental clinicians also reported instances where they received the necessary guidance from dental laboratory technicians, which contributed to treatment success.

D3: “He [technician] told me, ‘you have to go deeper with your margin.’ He enriches me with information I never even knew I needed. It is a win-win situation. Even as a dentist, I learn from him [technician].”

### Theme 2. Alignment of expectations

Participants from both groups highlighted the importance of mutual understanding regarding associated costs of laboratory work, turnaround time, and patient preferences. The importance of clear communication of expectations between clinicians and laboratory technicians, from the outset, was highlighted. This, in turn, helps optimize patient care and saves effort and resources. Clear and realistic expectations indicated a higher level of satisfaction for both groups.

Laboratory technicians reported an underestimation of the time required to complete cases and the lengthy steps involved, especially by novice clinicians and dental students. Some technicians reported rejecting cases with “fast track” requests due to the unrealistic turnaround time set by the clinician or their patients.

Participants stressed that communicating the estimated time and the number of appointments needed to complete a procedure was perceived as the clinicians’ responsibility. Technicians highlighted the need for dental clinicians to be more rigid about setting boundaries and not respond to unrealistic time demands from patients.

LT6: “Some patients think we have a magic wand to prepare the prosthesis. The quality of rushed work would never be as good as that of unrushed cases.”

A major reason for frustration expressed by both groups was the recurrence of unexpected delays due to slow production, remakes, or repairs, resulting in loss of revenue and clinical time and compromising quality of patient care. Dental laboratory technicians attributed these issues to insufficient information or poor-quality records provided by clinicians.

LT4: “The dentist should add more detailed information beforehand. Unfortunately, this is where the remake begins.”

Dental clinicians reported that the repetition of impressions is indeed stressful and challenging, especially in patients with limited mouth opening or uncooperative children. However, they felt that laboratory technicians sometimes lack empathy when asking the clinicians to resend better quality work.

D6: “My record time was eight times; I had to repeat the impression for a single crown.”

Participants emphasized that a clear understanding of patient preferences, especially those related to cosmetics and shade selection, was important for patients’ acceptance of the final treatment. Some technicians highlighted that proper expectation alignment with patients begins by establishing a patient-technician rapport through personal meetings to discuss patients’ esthetic preferences. This would help personalize the treatment and fabricate the desired appliances while accounting for a patient’s character and face shape.

### Theme 3. Teamwork dynamics

Participants valued one-to-one relationships rather than transactional ones. They emphasized that mutual trust, respect, and understanding nourished relationships and helped transform them from work-based to more personal camaraderie and friendship over time.

LT1: “The laboratory is not a post office where you drop and collect the cases. Even pro-technicians cannot do great work without communication.”

D5: “There is a human factor; the laboratory sent a thank you card and a candy with the case.”

Participants appreciated “working together” as a team towards a shared goal of providing patients with high-quality dental treatment. Long-term relationships created a sense of loyalty between clinicians and technicians because the two parties understand each other’s preferences, resulting in a smoother and “harmonious” interaction.

D4: “They [the clinician and laboratory technician] have this compatible, symbiotic relationship that made the result of the crown almost perfect. Their relationship was for 30 years. It wasn’t a 2- or 3-year kind of relationship.”

D6: “I have worked with a technician for 4 to 5 years, and we have this harmony going on. We have developed a system. That is why I no longer send my cases outside. We taught each other. He has his input, and I have mine. We have reached a middle ground where we understand each other”.

The dental technicians felt that dental clinicians are the leaders in this relationship, and they are expected to be empathetic listeners to both the patients’ demands and the technicians’ input.

LT2: “They [clinicians] are the brain, and we [laboratory technicians] are the muscle.”

Technicians who felt left out of treatment planning for some cases, highlighted that their involvement made them feel valued as members of the dental team and more engaged. Furthermore, the implementation of their suggestions made them feel confident.

Participants appreciated when the other party was receptive to feedback on their work. They expressed that admitting mistakes reflected their self-accountability and increased their respect, strengthening their professional relationships.

D3: “I think our responsibility as dentists is to help and guide dental laboratory technicians and not to blame them. The problem is the blame game we play with laboratory technicians.”

Laboratory technicians reported being criticized for their laboratory work in front of the patient in many instances and described these incidents as “unprofessional” and “unethical”. This was detrimental to technician confidence in their skills. In contrast, clinicians who acknowledged laboratory technicians in front of patients were perceived as “leaders.”

LT10: “When the doctor calls me to the clinic to thank me, to make me see the smile I created for the patient, I become extremely happy and grateful.”

Perceived hierarchy and status differentials were mentioned as the main barriers to the efficiency of teamwork dynamics. Some technicians felt discouraged from providing clinicians with feedback and believed that they were resistant to listening to the advice given by the technicians.

### Theme 4. Means of communication

Technicians appreciated the use of visual aids including drawings or images. These were specifically utilized for designing removable prosthodontic and orthodontic appliances. Some dental clinicians and dental laboratory technicians favored verbal discussions in the form of face-to-face or virtual audiovisual calls to supplement written prescriptions, especially at the beginning of a relationship.

D4: “I prefer the old-fashioned way over the digitized way where the dentist and the technician do not have an established relationship. We need to prioritize building that relationship rather than digitizing it.”

LT4: “The dentist should visit the laboratory himself before sending his or her cases to start building up the communication. Talk to the laboratory staff personally, understand the skills available, and explain his way of work.”

Communication during dental cosmetic cases seemed to be a major issue. Both clinicians and technicians felt that technician access to the clinic is important to ensure proper selection of shape and translucency level based on a patient’s overall look and preferences. Hence, clinicians and laboratory technicians favored in-house laboratories over remote ones. This was attributed to the ability to build a one-to-one relationship.

D2: “We do not communicate well with technicians because they are not in our clinic. If the technicians worked in the same clinic, it would be easier for them to see the patient clinically, and I can explain everything.”

Many technicians felt that they often receive “minimum information” in the prescription, which can affect the workflow and quality of the final results. Technicians who fabricated veneers emphasized the need for extra- and intra-oral pretreatment photographs, study models, and precise jaw relation records to ensure the design and fabrication of optimal cosmetic work.

### Theme 5. Operational management

Clinicians discussed the shortage of fully equipped laboratories, limited number of talented ceramists, and specialized laboratory technicians for fabricating removable orthodontic appliances and clear aligners. The low laboratory-to-clinic ratio in some regions was a major concern, resulting in an inability to meet demands. Consequently, some dental clinics routinely outsourced their lab work to national and international laboratories despite the high costs. Participants highlighted that despite these laboratories being remote, they had the competitive advantage of being equipped with advanced digital communication systems and workflow.

Dental laboratory technicians felt the need for close management, supervision, quality control checks, and effective handling for the high demands from dental clinics. The lack of incentives and limited training opportunities were among the issues discussed by technicians, which caused poor job satisfaction, resulting in a loss of motivation or even career alteration. Additionally, dental laboratory technicians felt that the management system must ensure a fair workload to prevent overloading technicians, which can result in an increased chance of systematic errors and poor overall quality of work.

Participants discussed the value of a good management system for clinics and laboratories to ensure good work relationships and profitable businesses. Some dental laboratories have implemented quality control units with several checkpoints to ensure the conditions of cases received or sent met certain standards. This was believed to help maintain relationships with the dental clinicians. Participants claimed that patients are usually the “victims” of management-related issues.

Some management systems do not allow one-to-one relationships. Examples included laboratories with an assembly line of technicians in which more than one technician performs the work. Furthermore, some laboratories do not permit clinicians from accessing the laboratory to prevent work interruptions caused by direct communication.

Dental laboratory owners agreed that the pricing strategy for laboratory services is challenging because various factors must be considered to keep the business operational and profitable. The competitive lowering of service prices is a common strategy used by new and small dental laboratories to attract clinic owners. However, both dental clinicians and laboratory technicians highlighted the negative effects of lowering prices on the quality of work delivered and the harm caused to high-quality laboratories.

D6: “There is fierce competition between laboratories. Every laboratory tries to lower its prices, and it is basically a race to the bottom. This compromises the quality of work.”

Clinicians also highlighted the issue of how dental laboratory technicians have slowly adopted digital dentistry. Furthermore, laboratory managers explained that small and medium businesses do not have the capital to invest in expensive technologies when cheaper labor is more cost-effective.

One of the challenges faced by dental laboratories was financial remuneration. According to the participants, it is common for dental clinics to suffer from accumulated debts and delays in payment. As a result, the completed cases are held by laboratory management. This places clinicians in a difficult situation with the patient and negatively affects the relationship between clinicians and technicians. Therefore, many laboratories are forced to terminate business relationships, which causes inconvenience to all parties involved.

LT6: “We used to contact the manager of one clinic to collect the debt, but no one responded. Meanwhile, they continued sending us more cases, and their debt increased. We had to stop working until we collected our debt; we struggled with them for about six months and will never work with them again.”

## Discussion

The current study highlighted the multitude of factors influencing the interprofessional relationship between dental clinicians and laboratory technicians at individual, interpersonal, and organizational levels. Perspectives from both groups were considered to capture rich insights into those factors. A one-to-one relationship with open communication channels and mutual trust and respect influenced partnership success. Demonstrating vulnerability by admitting mistakes and accepting feedback were desirable traits. Furthermore, the veracity of prescriptions and aligned expectations in relation to turnaround time and cost of laboratory work were prerequisites for successful treatment. Based on the current study’s findings, technician empowerment can be achieved through their direct involvement in decision-making and acknowledgment of their role in the presence of patients. The issue of technicians being “invisible” to patients was underscored in the interviews. This finding is in line with previous research, in which less than 10% of dental laboratory technicians involved in fixed prosthesis fabrication interacted with patients regularly [12].

Unsuccessful collaboration between dental clinicians and laboratory technicians can lead to work inefficiencies that may ultimately translate into suboptimal patient care. Interprofessional interventions encompass a range of activities in practice, organization, and education levels [21]. Therefore, to minimize the communication gap among dental teams, several approaches have been suggested to establish and nourish teamwork collaboration including organizing ice-breaking events, team bonding activities or games, informal meetings, and study clubs [22]. One study found positive short-term effects for the use of escape rooms on perceived cohesion in an interprofessional healthcare team [23]. Regarding improving operational procedures, adherence to quality control measures and best practices are likely to optimize work efficiency, reducing the margin of error and avoiding the associated lost costs [16]. Furthermore, frequent clinical and laboratory audits are important in order to ensure the latest procedures and standards are implemented [24]. Managers of dental clinics and/or laboratories could establish checklists and test their applicability for all transactions between the two entities to enhance the quality of delivered treatment [25]. Full automation and digitization of dental laboratories will inevitably increase precision, accuracy, and efficiency of laboratory procedures despite their high cost, and required training and skillsets [26].

Interprofessional education (IPE) has been suggested as a shared learning method to “bridge the gap” between different healthcare professionals working in the same team to improve patient care [27–29]. The core competencies of IPE include collaborative practice, interprofessional communication, and team-based care [27]. The independent structuring of dentistry and dental technology undergraduate programs has been a major concern [28]. Trainee dentists have limited exposure to dental laboratories, and trainee dental laboratory technicians have limited access to dental clinics [28]. There is evidence to suggest that pairing dental students with trainee dental technicians for the provision of dentures results in positive perceptions in aspects including communication, teamwork, and enhanced understanding of roles of the other profession [30, 31]. Furthermore, a comparative study including traditional and IPE curricula revealed significant benefits for dental technology students including the self-reported acquisition of team working and communication skills [32]. Hence, implementing such innovative IPE programs is encouraged, and more evidence is needed to support the effectiveness of different delivery types and timing of IPE in the dental curriculum [30].

Continuous professional development (CPD) is essential for both professional groups to further their skills and introduce them to new technology. CPD can be an opportunity to enhance teamwork interaction and understanding among dental team members [22]. A previous study reported issues concerning affordability and scarcity of courses targeting dental technicians [33]. Furthermore, a survey-based study reported that most training received by dental technicians on computer-aided design and computer-aided manufacturing technology was mainly company-led rather than provided by educational institutions [34], which poses a risk of potential misguidance in clinical practice by the involved companies. Consequently, employers need to consider the sponsorship and endorsement of such learning driven events.

Technological advancements have resulted in an industrial transformation in dental laboratories, exemplified by the ability to send digital scans for appliances or prostheses fabrication [26]. This has resulted in global competition among dental laboratories, especially with the ease of worldwide shipping, lower costs from foreign laboratories, and adequate quality of dental work [26]. In the current study, dental clinicians reported slow adoption of digital technology in some laboratories, which resulted in outsourcing complex prosthodontic and orthodontic cases treated with clear aligners. The reported disadvantages of outsourcing cases included difficulty in prescription interpretation, especially with a lack of face-to-face communication, in addition to the lack of clear information concerning the materials to be used and quality control procedures implemented [35]. Furthermore, reliance on foreign laboratories negatively affects the domestic dental technology industry, especially small to medium-sized laboratories [26, 36]. Therefore, the pressure is greater on domestic small to medium-sized laboratories to adopt technology more efficiently, which is usually hampered by their limited budget, to maintain their position in the market [26].

The complexity of understanding the factors influencing professional interpersonal relationships suggests that it is best addressed using qualitative methodology. In the current study, interviews rather than focus groups were used to allow participants to voice their concerns and reflect on their own experiences without being influenced by the opinions of others, especially those from the other profession. Participants' views may potentially be influenced by response bias. However, the interviewers used non-leading questions to enable participants to freely express their experiences. Snowball sampling was employed and the interviews were conducted virtually, to facilitate inclusion of participants from dispersed geographical locations [37]. The involvement of a heterogeneous sample enabled a holistic understanding of the relationship from the perspectives of different dental clinician and technician specialties. It also enabled an understanding of problems in different work sectors, including private clinics and university and non-university hospitals, and perspectives of clinic and/or laboratory owners. The number of participants included in the current study was sufficient to achieve data saturation, and was similar to the reported median sample size in previous qualitative studies in dentistry [38]. The interviewers had different professional backgrounds (restorative dentist and orthodontist), and both were involved in the data collection and analysis to mitigate preconceived ideas and potential biases. The focus of the current study was on the interrelationship between dental clinicians and technicians based in Saudi Arabia. Therefore, the transferability of the findings to other members of the dental team and different contexts is questionable. It is worth noting that there is limited data concerning the practices and training of dental laboratories in Saudi Arabia. Therefore, future research investigating these aspects is worth considering. Findings from the current study can be utilized to generate questionnaire items, as well as to inform the development of interventions directed at improving the interrelationship among members in the dental team.

## Conclusions

Five key factors influencing the dental clinician-laboratory technician interrelationship were identified from both perspectives, including educational background, alignment of expectations, means of communication, teamwork dynamics, and operational management. Considering the views of dental clinicians and laboratory technicians provided a holistic understanding of their issues at individual, interpersonal, and organizational levels, which can serve as a basis for initiatives and action plans focused on fostering harmonious relationships. It appears that revamping dental programs is necessary to implement interpersonal education and the rapid technological transformations in the industry.

## Data Availability

The data are available from Eman H. Ismail on reasonable request.

## Abbreviations

CPD: Continuous professional development
IPE: Interprofessional education

## References

[1] Ali SAA, Khalifa N, Alhajj MN (2018). Communication between dentists and dental technicians during the fabrication of removable partial dentures in Khartoum state Sudan. Acta Stomatol Croat.

[2] Haj-Ali R, Al Quran F, Adel O (2012). Dental laboratory communication regarding removable dental prosthesis design in the UAE. J Prosthodont.

[3] Hatzikyriakos A, Petridis HP, Tsiggos N, Sakelariou S (2006). Considerations for services from dental technicians in fabrication of fixed prostheses: a survey of commercial dental laboratories in Thessaloniki. Greece J Prosthet Dent.

[4] Tulbah H, AlHamdan E, AlQahtani A, AlShahrani A, AlShaye M (2017). Quality of communication between dentists and dental laboratory technicians for fixed prosthodontics in Riyadh Saudi Arabia. Saudi Dent J.

[5] Afsharzand Z, Rashedi B, Petropoulos VC (2006). Communication between the dental laboratory technician and dentist: work authorization for fixed partial dentures. J Prosthodont.

[6] Juszczyk AS, Clark RK, Radford DR (2009). UK dental laboratory technicians’ views on the efficacy and teaching of clinical-laboratory communication. Br Dent J.

[7] Azzopardi L, Zarb M, Alzoubi EE (2020). Quality of communication between dentists and dental laboratory technicians in Malta. Xjenza.

[8] Lynch CD, Allen PF (2005). Quality of communication between dental practitioners and dental technicians for fixed prosthodontics in Ireland. J Oral Rehabil.

[9] Owall B (1974). Design of removable partial dentures and dental technician education. Sven Tandlak Tidskr.

[10] Leeper SH (1979). Dentist and laboratory: a “love-hate” relationship. Dent Clin North Am.

[11] Scott H (1983). The lab and the dentist: enemies or friends?. Dent Lab Rev.

[12] Berry J, Nesbit M, Saberi S, Petridis H (2014). Communication methods and production techniques in fixed prosthesis fabrication: a UK based survey. Part 1: communication methods. Br Dent J.

[13] Bower EJ, Newton PD, Gibbons DE, Newton JT (2004). A national survey of dental technicians: career development, professional status and job satisfaction. Br Dent J.

[14] Berry J, Nesbit M, Saberi S, Petridis H (2014). Communication methods and production techniques in fixed prosthesis fabrication: a UK based surve Part 2: production techniques. Br Dent J.

[15] Oancea L, Panaitescu E, Burlibasa M, Gagiu C (2022). Clinical versus dental laboratory survey regarding modern fixed implant supported prosthetic in Romania. Appl Sci.

[16] Metz MJ, Abdel-Azim T, Miller CJ, Lin WS, ZandiNejad A, Oliveira GM, Morton D (2014). Implementation of a laboratory quality assurance program: the Louisville experience. J Dent Educ.

[17] Corcodel N, Zenthöfer A, Setz J, Rammelsberg P, Hassel AJ (2011). Estimating costs for shade matching and shade corrections of fixed partial dentures for dental technicians in Germany: a pilot investigation. Acta Odontol Scand.

[18] Kumar S, Mohammadnezhad M (2022). Perceptions of dental health professionals (DHPs) on job satisfaction in Fiji: a qualitative study. BMC Health Serv Res.

[19] Naderifar M, Goli H, Ghaljaie F (2017). Snowball sampling: a purposeful method of sampling in qualitative research. Strides Dev Med Edu.

[20] Ritchie J, Lewis J, Nicholls CMN, Ormston R. qualitative research practice: a guide for social science students and researchers. SAGE Publications; 2013.

[21] Reeves S, Goldman J, Gilbert J, Tepper J, Silver I, Suter E, Zwarenstein M (2011). A scoping review to improve conceptual clarity of interprofessional interventions. J Interprof Care.

[22] Christensen GJ (2009). Improving dentist-technician interaction and communication. J Am Dent Assoc.

[23] Cohen TN, Griggs AC, Kanji FF, Cohen KA, Lazzara EH, Keebler JR, Gewertz BL (2021). Advancing team cohesion: Using an escape room as a novel approach. J Patient Saf Risk Manag.

[24] Stewart CA (2011). An audit of dental prescriptions between clinics and dental laboratories. Br Dent J.

[25] Bresciano ME, De Maria A, Morello M, Poglio E, Audenino G (2017). Efficacy of a checklist for office-laboratory communication: a clinical study on quality outcomes for single crowns. Int J Prosthodont.

[26] Kneissl L, Modre C. Dental laboratory crisis: how is Chinese competition affecting the Swedish dental industry? Master’s Thesis, Jönköping University*.* 2017.

[27] World Health Organization. Framework for action on interprofessional education and collaborative practice. Geneva, (No. WHO/HRH/HPN/10.3); 2010.

[28] Reeson MG, Jepson NJ (2005). “Bridging the gap.” Should the training of dental technicians be linked with that of the dental undergraduate?. Br Dent J.

[29] Reeves S, Perrier L, Goldman J, Freeth D, Zwarenstein M (2013). Interprofessional education: effects on professional practice and healthcare outcomes (update). Cochrane DB Syst Rev.

[30] Reeson MG, Walker-Gleaves C, Jepson N (2013). Interactions in the dental team: understanding theoretical complexities and practical challenges. Br Dent J.

[31] Reeson MG, Walker-Gleaves C, Ellis I (2015). Attitudes towards shared learning of trainee dental technicians and undergraduate dental students. J Dent Educ.

[32] Evans JL, Henderson A, Johnson NW (2013). Traditional and interprofessional curricula for dental technology: perceptions of students in two programs in Australia. J Dent Educ.

[33] Anderson VR, Pang LC, Aarts JM (2012). New Zealand dental technicians and continuing education: findings from a qualitative survey. N Z Dent J.

[34] Blackwell E, Nesbit M, Petridis H (2017). Survey on the use of CAD-CAM technology by UK and Irish dental technicians. Br Dent J.

[35] Christensen GJ, Yancey W (2005). Dental laboratory technology in crisis: the challenges facing the industry. J Am Dent Assoc.

[36] Alameri SS, Aarts JM, Smith M, Waddell JN (2014). Dental technology services and industry trends in New Zealand from 2010 to 2012. N Z Dent J.

[37] Parker C, Scott S, Geddes A. Snowball Sampling, In P. Atkinson, S. Delamont, A. Cernat, JW. Sakshaug, RA. Williams (Eds.), SAGE Research Methods Foundations. SAGE Publications Ltd; 2013. 10.4135/9781526421036831710.

[38] Al-Moghrabi D, Tsichlaki A, Alkadi S, Fleming PS (2019). How well are dental qualitative studies involving interviews and focus groups reported?. J Dent.

